# Reported Māori consumer experiences of health systems and programs in qualitative research: a systematic review with meta-synthesis

**DOI:** 10.1186/s12939-019-1057-4

**Published:** 2019-10-28

**Authors:** Suetonia C. Palmer, Harriet Gray, Tania Huria, Cameron Lacey, Lutz Beckert, Suzanne G. Pitama

**Affiliations:** 1 Department of Medicine, University of Otago Christchurch, 2 Riccarton Ave, Christchurch, 8140 New Zealand; 2 Māori and Indigenous Health Institute, University of Otago Christchurch, 45 Cambridge Terrace, Christchurch, 8140 New Zealand

**Keywords:** Indigenous, Health services, Quality, Synthesis, Systematic review

## Abstract

**Background:**

Persistent inequities in health experiences and outcomes are observed for Māori compared to non-Māori in Aotearoa New Zealand. We conceptualised factors associated with Māori consumer experiences of health programs and services and characterise how the recommendations arising from qualitative research inform strategies to address inequities.

**Methods:**

In this systematic review, electronic literature searching was conducted in February 2018. Qualitative studies reporting Māori consumer experiences of health services and programs in Aotearoa New Zealand were eligible. Māori consumer experiences of health services were mapped to the WHO Commission of Social Determinants of Health (CSDH) conceptual framework on health inequities as related to: (i) the socioeconomic and political context; (ii) socioeconomic positioning; or (iii) intermediary factors that increase exposure to health-compromising conditions. Recommendations to improve consumer experiences were mapped to the CSDH framework for tackling social determinants of health inequities as policy directions on: (i) unequal consequences of illness (individual interaction); (ii) risks of exposure to health-damaging factors (community); (iii) exposures to health-damaging factors (public policies); and (iv) mitigating effects of socioeconomic and political stratification (environment).

**Results:**

Fifty-four studies were included. Māori consumer experiences mapped to social determinants of health inequities were most frequently related to direct interactions with health services and programs, particularly patient-clinician interactions (communication, relationships) and cultural competencies of clinicians and the system. Key recommendations by researchers mapped to potential strategies to address inequity were identified at all levels of the political, social and health system from individual interactions, community change, and broader public and system-level strategies. Recommendations were predominantly focused on actions to reduce risks of exposure to health-damaging factors including health literacy interventions, increased resources in cultural competencies and Māori capacity in health service development and workforce.

**Conclusions:**

Māori consumer experiences of health services and programs are an important informer of variables that impact health inequity. Strategies to tackle health inequities informed by Māori consumer experiences can be drawn from existing empirical research. Future qualitative exploration of how socioeconomic, political and public policies influence Māori consumer experiences of health services and programs could inform a broader range of structural policies to address health inequities.

## Background

Persistent and marked inequity is observed for Māori at all levels of health, [1] education (Ministry of Health, Ministry of Education: Māori participation and attainment in science subjects, unpublished) and justice [2] in Aotearoa New Zealand. Non-Māori have life expectancies approximately 7 years longer than Māori, attain higher educational achievement in secondary and tertiary education, and are incarcerated at markedly lower levels. In primary and secondary health care, non-Māori patients are prescribed more effective medications [3], are referred more often to specialist services [4] and experience higher quality hospital care [5]. Non-Māori patients experience persistently lower rates of preventable diseases that lead to avoidable hospitalisation and unmet need in primary care [6]. Māori experience inequitable access to health services throughout the life course leading to higher rates of disability and multiple morbidity [7]. Māori are more likely than non-Māori to cite cost as a barrier to accessing primary care. In addition, nationwide quality improvement programs in Aotearoa New Zealand worsen inequity by differentially improving access to services for non-Māori [8–10].

Patient-centred research can facilitate the understanding of consumer experiences, perceptions and expectations of health services to generate insights and knowledge that guide improvements in healthcare acceptability and quality [11]. In the last two decades, there has been an increase in qualitative research to explore patient viewpoints to inform public policy and align health service development with consumer preferences and expectations [12]. Despite this shift toward greater inclusion of patient voices in clinical health research, including with Māori consumers, health outcomes remain inequitable across numerous clinical settings in Aotearoa New Zealand and for indigenous and tribal peoples worldwide [13]. In addition to seeking patient perspectives, qualitative studies can offer critical insights into the ways that researchers view and conceptualise the patient experience and how those experiences are problematised as a basis for interventions to improve health outcomes [14, 15].

This study aimed to explore how Māori consumer experiences of health services and programs in Aotearoa New Zealand are conceptualised within qualitative research, to characterise how recommended strategies to improve Māori consumer experiences can inform policy directions to address health inequities and to identify gaps in the existing evidence base.

## Methods

We did a systematic review and evidence synthesis of qualitative studies reporting Māori consumer experiences of health services and programs in Aotearoa New Zealand. We used the theoretical framework of the World Health Organization (WHO) Commission of Social Determinants in Health (CSDH) to categorise the factors reported to be associated with Māori consumer experiences of health and to evaluate how recommended strategies arising from the findings might inform strategies to address health inequities [16]. The Enhancing Transparency in Reporting the Synthesis of Qualitative Research (ENTREQ) framework was followed for this study [17].

### Literature searching

Electronic searches were conducted in AMED, CINAHL, EMBASE, MEDLINE, Google Scholar, PROQUEST, and PsycINFO for qualitative studies reporting Māori consumer experiences of health services and programs in Aotearoa New Zealand from each database inception up to week 2, February 2018. We used search terms using keywords relating to Māori (Māori, First nation, Oceanic ancestry, Native), and qualitative research (Content analysis, Descriptive, Discourse, Exploratory, Grounded theory, Interpretive, Interview, Mixed method, Multi method, Narrative, Phenomenology, Qualitative, Thematic, Theme) and experiences (Attitude, Belief, Experience, Perception, Perspective, Satisfaction, Value, View). Studies that included Māori participants but that did not provide separate data analysis for Māori and non-Māori participants were not eligible.

### Data extraction and quality assessment

The following basic characteristics were extracted from each study: publication year, methods for ethnicity determination, number of Māori participants, gender, cohort characteristics, health setting, topic, study methodological framework, whether kaupapa Māori methodologies were used and funding source(s). The text of each paper including tables and figures was reviewed in full text by at least two of three authors (SCP, HG, SP) to extract the following from each study: stated purpose of research, determinants of Māori participant experiences, and research responses and recommendations arising from the findings. Two authors independently assessed the transparency of reporting using the Consolidated Criteria for Reporting Qualitative Research (COREQ), which assessed study methodological reporting of the research team, methodologies, context, analysis, and interpretation [18].

### Data coding and analysis

The extracted data underwent two cycles of coding by at least two of three authors (SCP, HG, SP). These authors (SCP and SP) have expertise in Māori health research or (HG) are training in Māori health research. Three authors who provided intellectual feedback on the coding also have expertise in Māori health research (TH, CL, LB).

In the first coding cycle, descriptive coding was used to identify the basic topic for each passage of reported data in the results and discussion section of each included study [19]. In the second cycle of coding, the determinants of Māori consumer experiences of the health system (services and programs) were mapped against the World Health Organization Commission for Social Determinants of Health (CSDH) conceptual framework of the determinants, processes and pathways that generate health inequities [16]. The CSDH conceptual framework includes the socioeconomic and political context in which people live (governance, macroeconomic policies, social and public policies and culture and societal values), the socioeconomic positioning of people (social class, gender, racism, education, occupation and income) and the intermediary factors (material circumstances, behaviours and psychosocial factors) which, mediated through health services and programs, determine inequity in health and wellbeing. The actions recommended by researchers arising from the determinants of Māori consumer experiences were mapped against the CSDH framework for strategies tackling social determinants of health inequities. This framework describes a hierarchy of dimensions and directions for: 1) policies to reduce unequal consequences of illness in social, economic and health terms (individual interactions); 2) policies to reduce risk of exposure of disadvantaged people to health-damaging factors (community); 3) policies to reduce exposures of disadvantaged people to health-damaging factors (public policies); and 4) policies on stratification to reduce inequalities and mitigate effects of stratification (environment).

## Results

**Fig. 1:**
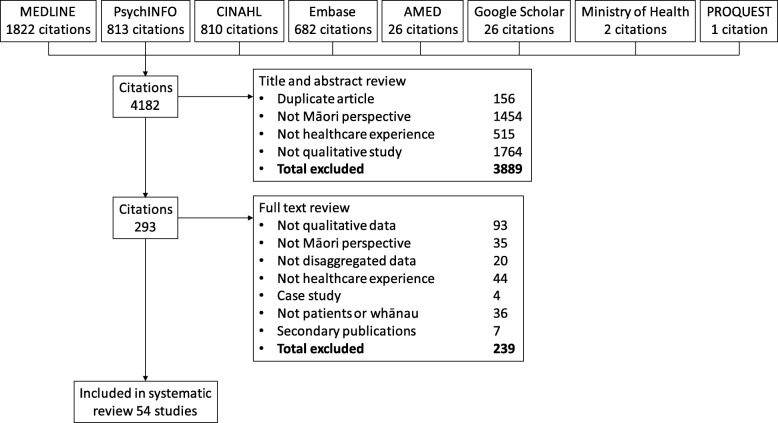
Study selection

Electronic searching yielded 4182 citations of which 293 were examined in full text (Fig. 1). Fifty-four qualitative studies were included [12, 20–72]. The comprehensiveness of study reporting is shown in Additional file 1 and in the Additional file 2. Studies reported between 4 and 26 of the 32 Consolidated Criteria for Reporting Qualitative health Research (COREQ) criteria.

Māori consumer experiences were reported within a range of health settings and programs including primary care, public health and screening initiatives, community health programs, disability and hospice services and hospital-based care (Table 1). The number of Māori participants in each study ranged between 4 and 130. Twenty-eight studies reported funding from government sources. Eleven studies reported kaupapa Māori (Māori-led) methodologies [12, 24, 35, 39, 56, 59, 61, 65–67, 70]. In 24 studies, ethnicity was reported as self-identified by participants [26, 27, 31, 34, 35, 37, 40, 45, 47–50, 55, 57, 59, 61, 63, 65–70, 72]. In two studies, ethnicity was identified through name or records, [29, 56].

**Table 1:** Characteristics of included studies

Study	Participants	Ethnicity determination	N	Age range	Male: Female	Health setting	Topic	Methodological framework	Funding source	Kaupapa Māori
White et al., 1999 [20]	Ngāti Tama marae iwi members	…	19	14–60 years	…	Physiotherapy	Perceptions of health, illness and physiotherapy among Maori	…	HRC	…
Tipene-Leach et al., 2000 [21]	Parents or caregivers of under 12-month-old infants within Maori, Tongan, Samoan, Cook Island, Niuean, and Pākehā communities	…	26	Mid-teens to late thirties	9:17	Sudden infant death syndrome	Infant care practices	…	HRC	…
Bassett and Tango, 2002 [22]	Patients recently completed or undergoing physiotherapy	…	6	…	1:5	Physiotherapy	Experiences of physiotherapy	Phenomenology	HRC	…
Buetow et al., 2003 [31]	Consumer representatives	…	10	30–79	3:7	General practice	Accessing general practitioner care for child asthma	…	…	…
Cram 2003 [24]	Members of urban, marae-based health services	…	28	17–75	…	Marae-based health program	Māori health	Kaupapa Māori	HRC	Yes
Williams 2003 [25]	Men with abnormal blood test (prostate-specific antigen)	…	20	40–70	20:0	Prostate disease	Health seeking for prostate health problems	Naturalistic approach	HRC; Wellington Medical Research Foundation; Wellington School of Medicine Surgical Research Trust; The Community Trust of Wellington; The University of Otago	…
Gibbs 2004 [26]	Patients who had been under community treatment order	Self-identification	8	27–50	6:2	Community treatment orders in psychiatric care	Community treatment orders	…	HRC	…
Glover 2005 [27]	Smokers intending to quit	Self-identification	130	16–62	29:101	Smoking cessation	Smoking cessation	Te Whare Tapa Wha	HRC	…
van der Oest 2005 [28]	Community members	…	…	…	…	Community groups	Health services for tuberculosis	…	…	…
Bolitho 2006 [29]	Whānau accessing care for child with respiratory illness	Health record	4 families	…	…	Paediatric hospital care	Accessing care for children with respiratory illness	…	…	…
Corbett 2006 [30]	Whānau of patients experiencing stroke	…	7 focus groups/3 interviews	…	…	Hospital care for stroke	Whānau experiences of caring for whānau with stroke	…	…	…
Penney 2006 [12]	Patients with ischaemic heart disease	…	25	40–70+	14:11	Primary health care	Heart disease management	Kaupapa Māori action research	HRC	Yes
Buetow 2007 [31]	Women overdue for cervical smear	Self-identification	5	39–57	0:5	Cervical screening program	Cervical cancer screening	Hermeneutic phenomenology	National Screening Unit	…
Dew 2007 [32]	Patients in general practice	…	7	…	2:5	General practice	Mental health in general practice	…	University of Otago	…
Lovell 2007 [33]	Women overdue for cervical smear	…	4	…	…	Cervical screening program	Cervical cancer screening	…	…	…
Fernandez 2008 [34]	Women who had stopped smoking	Self-identification	5	28–45	0:5	Smoking cessation	Smoking cessation initiatives	…	…	…
Waetford 2008 [35]	Young women	Self-identification	16	17–23	0:16	Sexual health	Sexual health and sexually transmitted infections	Kaupapa Māori	HRC; Ministry of Health	Yes
Walker 2008 [36]	Patients and whānau affected by cancer	…	44	20–80	…	Cancer	Experiences of cancer	…	Cancer Society of NZ	…
Wilson 2008 [37]	Women	Self-identification	38	24–61	0:38	Health services	Mainstream health services	Grounded theory	…	…
Arlidge 2009 [38]	Whānau of children admitted to hospital for injury	…	8 whānau	…	…	Injured children	Whānau experiences of hospital care for injured children	…	HRC	…
Edwards 2009 [39]	Fathers of babies dying from SIDS	Self-identification	9	20–45	9:0	Sudden infant death syndrome	Experiences of sudden infant death syndrome	Kaupapa Māori; discourse analysis	…	Yes
Glover 2009a [40]	Mothers and whānau with babies born in previous 3 years	Self-identification	59 mothers; 27 whānau members	…	…	Infant feeding	Breastfeeding	…	HRC	…
Glover 2009b [41]	Takatāpui with experience of assisted reproduction/infertility	…	8	38–58	3:5	Assisted reproduction	Assisted human reproduction	…	…	…
Jansen 2009 [42]	Health consumers	…	86 (10 hui)	…	…	Health services	Health services	…	HRC; Ministry of Health; ACC	…
Wiley 2009 [43]	Consumers and caregivers using disability services	…	34	…	…	Disability services	Disability services	…	Fulbright NZ; Te Pūmanawa Hauora	…
Handley 2010 [44]	Patients with type 2 diabetes	…	4	…	…	Type 2 diabetes	Lived experiences of type 2 diabetes	Phenomeonological; grounded theory	General Practice Trust, the Ministry of Health Primary Health Care Nursing fund, the New Zealand Nurses Organisation, the Whanganui District Health Board Clinical Training Agency fund and Diabetes New Zealand	…
Laird 2010 [45]	Families in contact with mental health services	Self-identification	18	…	…	Mental health services	Diagnostic classification within mental health services	…	…	…
McManus 2010 [46]	Women experiencing sudden infant death syndrome	…	17	19–38	0:17	Sudden infant death syndrome	Life story of women experiencing sudden infant death syndrome	…	HRC	…
Pitama 2011 [47]	Patients using health services	Self-identification	30	25–70	11:19	Primary health care	Te reo in primary care	…	Ministry of Health	…
Shih 2011 [48]	Haemodialysis patients living in rural area	Self-identification	7	46–77	…	Haemodialysis	Experience of haemodialysis	Hermeneutical analysis	…	…
Thompson-Evans 2011 [49]	Smokers	Self-identification	18	16–60	7:11	Smoking cessation	Rongoa to support smoking cessation	…	University of Auckland	…
Bassett-Clarke 2012 [50]	Community members	Self-identification	21	50–84	6:15	Community	Medicine taking	Bio-psychosocial model	Pharmacy Education Research Foundation	…
Evans 2012 [51]	Community members	…	…	…	…	Community	Genetic technologies	Interpretive phenomenological analysis	…	…
Glover 2012 [52]	Pregnant women who smoke	…	60	17–43	0:60	Māori primary health care services	Smoking cessation in pregnancy	…	…	…
Gray 2012 [53]	Community members	…	28	…	…	Communities vulnerable to H1N1 virus infection	Communication campaigns for influenza A (H1N1)	…	HRC; Ministry of Health	…
Sheridan 2012 [54]	Patients admitted to hospital with chronic health condition	…	8	…	…	Chronic health conditions leading to hospital admission	Engagement with primary health care	…	New Zealand Tertiary Education Commission	…
Wilson 2012 [55]	Patients admitted to medical or surgical setting	Self-identification	11	…	…	Acute medical-surgical hospitalisation	Hospital experiences	…	HRC; Ministry of Health	…
Elder 2013 [56]	Marae communities	Identified by marae koroua, kuia, rōpū	9 marae	…	…	Child and adolescent brain injury	Child and adolescent brain injury	Kaupapa Māori	…	Yes
Frey 2013 [57]	Cancer patients, whānau (including bereaved whānau)	Self-identification	7	…	…	Hospice care	Hospice service use	…	Counties Manukau District Health Board; Totara Hospice South Auckland	…
Johnson 2013 [58]	Homeless people	…	6	…	3:3	Homelessness	Needs of homeless people with mental health concerns	…	…	…
Lee 2013 [59]	Sole mothers	Self-identification	7	…	0:7	Primary health care	Barriers to sole mothers’ access to primary care	Kaupapa Māori	University of Auckland	Yes
Slater 2013 [60]	Patients and whānau affected by cancer	…	12 patients and whānau	Mid-30s to mid-70s	…	Cancer care services	Access to and through cancer care	…	HRC; Ministry of Health	…
Te Karu 2013 [61]	Patients with gout	Self-identification	12	48–79	…	Gout treatment	Gout management	Kaupapa Māori	…	Yes
Angelo 2014 [62]	Family members of dying patients	…	2	38–59	1:1	Hospice care	Hospice family caregiving	…	…	…
Bakker 2014 [63]	Patients referred for CPAP treatment	Self-identification	25	…	…	Obstructive sleep apnoea treatment	Continuous positive airway pressure treatment for obstructive sleep apnoea	…	HRC	…
Johnston Taylor 2014 [64]	Family members of patients who had used hospice service and kaumātua	…	7	34–74	1:6	Hospice care	Hospice care	Kaupapa Māori	HRC	Yes
Makowharemahihi 2014 [65]	Pregnant woman pre- and after birth	Self-identification	44	14–20	0:44	Maternity care	Maternity care	Kaupapa Māori	HRC	Yes
McLellan 2014 [66]	Patients and whānau affected by aphasia	…	11 patients and 23 whānau members	50–79	4:7	Speech language therapy	Speech language therapy services	Kaupapa Māori	University of Auckland; Henry Rongomau Bennet scholarship; Tavistock Trust for aphasia	Yes
School of Physio 2014 [67]	Consumers of disability services and whānau	Self-identification	29	17–74	18:10	Disability services	Disability services	Kaupapa Māori	HRC; Ministry of Health	Yes
Abel 2015 [68]	Mothers	Self-identification	12	19–39	0:12	Infant sleeping	Wahakura (flax bassinet) use for infant sleeping	Māori-focused	New Zealand Lotteries Grants Board; Eastern Institute of Technology	…
Slater 2015 [69]	Hospice patients and whānau	Self-identification	8 patients and whānau	…	…	Hospice care	Hospice care	…	HRC	…
Warbrick 2016 [70]	Sedentary and overweight men	Self-identification	18	28–72	18:0	Physical activity	Physical activity and exercise	Kaupapa Māori	HRC	Yes
Tapera et al., 2017 [71]	Grandparents	…	5	…	…	Urban neighbourhood	Feeding of grandchildren	Kaupapa Māori-consistent approach	None	…
Walker et al., 2017 [72]	Patients nearing need for dialysis or started dialysis	Self-identification	13	20–80	–	Dialysis units	Chronic kidney disease	Grounded theory	Baxter Clinical Evidence Council; New Zealand Lotteries Health Research Grant; Kidney Health New Zealand	…

*HRC* Health Research Council of New Zealand. … not data available or none reported

Most (38 studies) studies aimed to evaluate consumer experiences and perceptions of healthcare and health service delivery. Fewer studies evaluated patient experiences as they related to healthcare implementation and policy (8 studies), [46, 52–54, 56, 67, 68, 70] cultural appropriateness of healthcare (6 studies) [39, 43, 47, 50, 66, 69] or to identify the causes and impact of health inequality (1 study) [36].

**Fig. 2:**
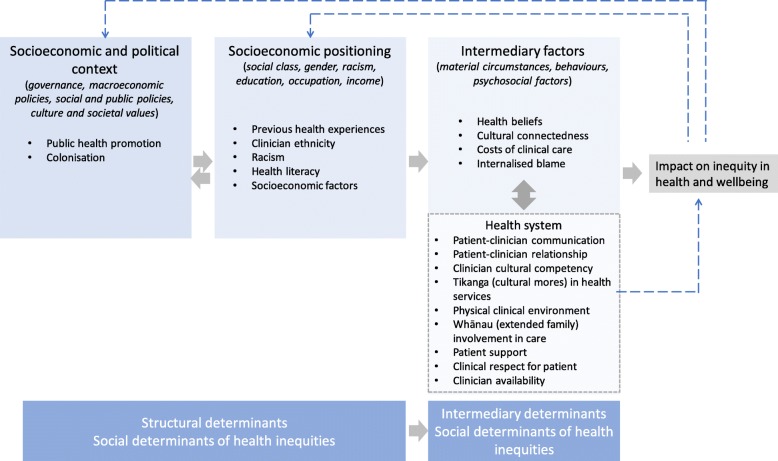
Reported Māori consumer experiences of Aotearoa/New Zealand health services and programs mapped to the Commission for Social Determinants of Health (CSDH) conceptual framework of health inequities [16]

Māori consumer experiences of health that were mapped to social determinants of health inequity were most frequently identified as direct interactions with the health system (Fig. 2). These included patient-clinician communication and relationships, clinician cultural competency, tikanga (cultural mores) in health services, the physical clinical environment, whānau (extended family) involvement in care, patient support, clinical respect for patient and clinician availability. Reported Māori consumer experiences mapped to intermediary factors that lead to differential exposures to health-damaging factors included health beliefs, cultural (dis) connectedness, costs of clinical care and internalised blame. Māori consumer experiences in the available studies were less commonly mapped to socioeconomic positioning (previous health experiences, clinician ethnicity, racism, health literacy and socioeconomic factors) and the socioeconomic or political context (public health promotion and colonisation).

**Fig. 3:**
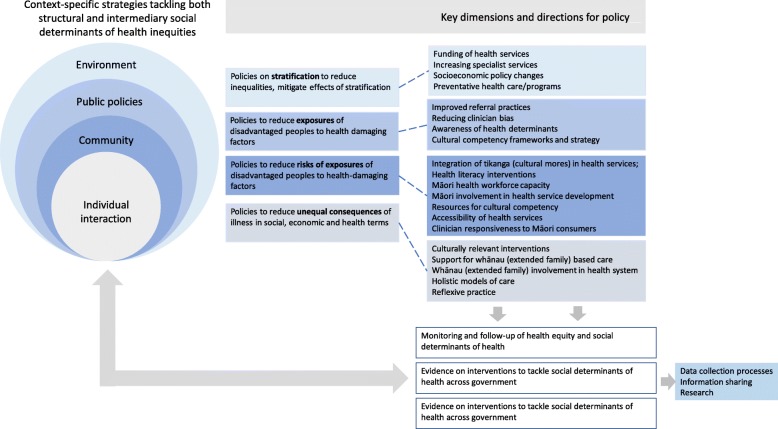
Researcher recommended actions to improve Māori experiences of Aotearoa/New Zealand health services and programs mapped to the Commission for Social Determinants of Health (CSDH) conceptual framework for tackling social determinants of health inequities [16]

Based on Māori consumer experiences, the most frequently recommended actions to improve Māori experiences of healthcare were aligned with *reducing risk of exposure to health-damaging factors* (such as integration of tikanga (cultural mores) in health services, health literacy interventions, increasing Māori workforce capacity and involvement in health service development, resources for cultural competency, accessibility of health services and clinician responsiveness to Māori consumers) (Fig. 3). Recommended actions to reduce the *unequal consequences of illness in social, economic and health terms* included culturally relevant interventions, support for whānau (extended family)-based care and involvement in the health system, holistic models of care and reflexive clinical practices. Strategies aimed at reducing *exposures to health damaging factors* included improved referral practices, reducing clinician bias, increased awareness of health determinants and provision of cultural competency frameworks and strategy. Proposed strategies aligned with *mitigating the effects of socioeconomic and political stratification* included funding of health services including increasing specialist services, socioeconomic policy actions and preventative health care and programs. Researchers also suggested mechanisms to monitor and follow-up on health equity based on Māori consumer experiences. These included data collection processes, information sharing and empirical health research (Fig. 3).

## Discussion

This paper summarises the determinants of Māori consumer experiences in health services and programs reported in qualitative studies and the actions recommended by researchers based on their findings. The findings have been mapped to the CSDH frameworks of health inequities to synthesise a range of potential strategies that might address Māori health inequities informed by consumer experiences. Based on qualitative data from several health settings, direct consumer interactions with the health system and programs are important informants of the determinants of health inequity in Aotearoa New Zealand, suggesting this as a priority area for quality improvement. Aspects of care that were particularly noted were patient-clinician relationships and communication, including clinician cultural competencies. Clinical services lacked alignment with tikanga (cultural protocols and processes) and involvement of the whānau in healthcare. Other intermediary factors that contributed to health inequity included costs of clinical care to consumers and internalised blame as a consequence of racism, leading to altered (reduced) care seeking actions. Systemic factors identified from exploration of Māori consumer experiences included socioeconomic and political factors such as colonisation, public health policies, institutional racism, power imbalances between clinicians and patients and health literacy.

Mapping of the strategies that researchers recommended to improve Māori consumer experiences to the CSDH framework for tackling health inequities enabled the CDSH framework to be aligned to Māori patient, whānau and consumer perspectives of the health system. Most recommendations provided strategies to reduce the risks of exposures to health-damaging factors experienced by Māori as the direct consequences of colonisation and racism. These responses included expanded use of tikanga and culturally competent practice in health services, capacity-building to support Māori participation in the health workforce and health service development, and greater access for Māori to clinical services including health system responsiveness to Māori consumer expressed needs and expectations. Health system level strategies based on consumer perspectives included greater funding of health services, expanding specialist services for Māori and increasing preventative health care and programs. Policies to reduce the unequal consequences of illness that further drive structural inequity included the development of culturally relevant interventions and whānau (extended family) rather than individual-centred care.

The actions identified by researchers that might reduce health inequities for Maori consumers in this synthesis are concordant with evidence within other national settings including Canada and Australia [73]. These include strengthening community-governed health services, addressing power imbalances during Indigenous patient interactions with health services through trust, reciprocity, and shared decision-making, as well as avoiding a deficit model of non-adherence by Indigenous patients as an explanation for health outcomes. Similarly, in a critical interpretive synthesis of healthcare in the United Kingdom among patients with socioeconomic disadvantage, equity of access was conceptualised as a complex interplay between social context and features of the health service such as patient navigation and the permeability of health services to specific patient communities [74]. These findings are consistent with the present analysis that identified potential strategies to address inequities and improve services for Māori consumers informed by experiences include modifying referral structures, and increasing service and clinician accessibility and responsiveness.

These findings suggest that, in addition to the role of qualitative research to evaluate individual and community-level Māori consumer experiences within specific health settings and encounters, there is an untapped potential for qualitative and participatory research with Māori consumers to inform the development and implementation of effective policies and interventions that reduce inequities and exposure to health damaging factors at a broader macro-level [75]. Aotearoa New Zealand has a governance system with the capacity to address health inequity as required by the Treaty of Waitangi. This system includes robust quantitative data collection and reporting on social determinants of health, legislative structures that enable intersectoral action on equity, a governmental framework linked to budget, and a strong public health system. While still relatively infrequent, qualitative research that is co-designed with Māori consumers has provided examples of how participatory research actions can inform system and policy-based change to address inequity. For example, research informed by Māori consumer and clinician experiences has led to consumer-designed health service improvements including increased specialist assessment and diagnostic services in rural Tai Tokerau/Northland [12] and a culturally-acceptable intervention to support safe bed-sharing (the wahakura sleeping pod), [21] that has subsequently been adopted as policy and evaluated in a randomised controlled trial [76, 77].

Clinical quality improvement programs in Aotearoa New Zealand do not always improve health service experiences and outcomes for Māori and may exacerbate rather than address inequities [8–10]. Recent examples include inequities in childhood immunisations that were nearly eliminated in 2014 but have re-emerged through progressive loss of initial gains in immunisation rates among Māori children [7]. Similarly, progressive improvements in diabetes monitoring and kidney disease screening for non-Māori have not occurred for Māori patients. Knowledge of Māori consumer experiences of health care to inform understanding of structural determinants and intermediaries of health inequities may support healthcare quality improvements that sustainably reduce the unequal distribution of quality health care. Empirical analysis has demonstrated the necessity of critical reflections of power and institutional culture in the sustainable delivery of programs that are aimed to impact on health inequity [78, 79].

The strengths of this paper include the *a priori* use of a conceptual framework that considers the structural determinants of health (CSDH), a broad literature search within multiple electronic databases and two levels of coding using established methodological processes. There are also limitations of this study that need to be considered when interpreting the findings. First, we may have not identified all the relevant qualitative studies available in the literature due to challenges in retrieval of qualitative research and a lack of searching of grey literature including governmental and non-governmental reports [80]. Second, we did not include qualitative studies exploring perspectives of health providers in the review which may have captured additional methodological approaches and theoretical frameworks in existing qualitative research of Māori consumer experiences. Third, the nature of qualitative research of patient experiences is frequently focused on the individual and their immediate family, and accordingly the type of study we evaluated would be most likely to examine the direct health consequences of unequal exposure to risk factors for disease and illness.

## Conclusions

Māori consumer experiences of health services and programs are an important informer of variables that impact health inequity. Strategies to tackle health inequities informed by Māori consumer experiences can be drawn from existing empirical research. Future qualitative exploration of how socioeconomic, political and public policies influence Māori consumer experiences of health services and programs could inform a broader range of structural policies to address health inequities.

## Supplementary information

**Additional file 1.** Comprehensiveness of study reporting by Consolidated Criteria for Reporting Qualitative health research.

**Additional file 2.** Dataset used for analysis.

## Data Availability

The dataset generated and analysed during the current study is available in the Mendeley Data repository, 10.17632/wgmwjscsn9.1.

## Abbreviations

COREQ: Consolidated Criteria for Reporting Qualitative Research
CSDH: Commission for Social Determinants of Health
ENTREQ: Enhancing Transparency in Reporting the Synthesis of Qualitative Research
