# Supplementary material for: Reported Māori consumer experiences of health systems and programs in qualitative research: a systematic review with meta-synthesis
Source: Int J Equity Health. 2019 Oct 28;18:163. doi: 10.1186/s12939-019-1057-4 (PMC6816189; doi:10.1186/s12939-019-1057-4)
Supplement: Supplementary file 1 — Additional file 1. Comprehensiveness of study reporting by Consolidated Criteria for Reporting Qualitative health research. [file 12939_2019_1057_MOESM1_ESM.docx]

## Additional File 1. Comprehensiveness of study reporting by Consolidated Criteria for Reporting Qualitative health research.

| **Reporting criteria** | **No. (%)** |
| --- | --- |
| **Domain 1: Research team and reflexivity** |  |
| **Personal characteristics (interviewer/facilitator)** |  |
| 1. Interviewer/facilitator | 32 (59%) |
| 1. Credentials | 20 (37%) |
| 1. Occupation | 24 (44%) |
| 1. Gender | 28 (52%) |
| 1. Experience and training | 6 (11%) |
| **Relationship with participants** |  |
| 1. Relationship established | 9 (17%) |
| 1. Participant knowledge of interview | 2 (4%) |
| 1. Interviewer characteristics | 25 (46%) |
| **Domain 2: study design** |  |
| **Theoretical framework** |  |
| 1. Methodological orientation and theory | 27 (50%) |
| **Participant selection** |  |
| 1. Sampling | 35 (65%) |
| 1. Method of approach | 37 (69%) |
| 1. Sample size | 51 (94%) |
| 1. Non-participation | 11 (20%) |
| **Setting** |  |
| 1. Setting of data collection | 20 (37%) |
| 1. Presence of non-participants | 17 (31%) |
| 1. Description of sample | 36 (67%) |
| **Data collection** |  |
| 1. Interview guide | 27 (50%) |
| 1. Repeat interviews | 4 (7%) |
| 1. Audio/visual recording | 42 (78%) |
| 1. Field notes | 7 (13%) |
| 1. Duration | 17 (31%) |
| 1. Data saturation | 12 (22%) |
| 1. Transcripts returned | 9 (17%) |
| **Domain 3: analysis and findings** |  |
| **Data analysis** |  |
| 1. Number of data coders | 27 (50%) |
| 1. Description of the coding tree | 19 (35%) |
| 1. Derivation of themes | 38 (70%) |
| 1. Software | 18 (33%) |
| 1. Participant checking | 11 (20%) |
| **Reporting** |  |
| 1. Quotations presented | 49 (91%) |
| 1. Data and findings consistent | 38 (70%) |
| 1. Clarity of major themes | 42 (78%) |
| 1. Clarity of minor themes | 8 (15%) |
